# Wellbeing and job satisfaction among employees with intellectual disability

**DOI:** 10.3389/fpubh.2025.1503932

**Published:** 2025-03-07

**Authors:** Andrew Joyce, Perri Campbell, Jenny Crosbie, Erin Wilson

**Affiliations:** Centre for Social Impact, Swinburne University of Technology, Melbourne, VIC, Australia

**Keywords:** job satisfaction, wellbeing, workplace, intellectual disability, health promotion

## Abstract

**Objectives:**

The workplace is considered one of the key settings in which to promote health and wellbeing. Reviews of workplace health promotion have shown that workplace interventions can positively impact on mental health, nutrition, and physical activity, and can impact positively on economic indicators such as absenteeism. One of the research gaps is workplace health promotion for people with an intellectual disability. This is an important gap to address as people with an intellectual disability have higher rates of avoidable mortality relative to the general population, increased rate of mental health problems, lower levels of physical activity, and poorer nutrition. People with an intellectual disability work across a range of industries and employment settings and it is important to understand potential strategies in supporting the health and wellbeing of this cohort within workplaces.

**Methods:**

Forty-seven in-depth interviews were conducted with staff and supported employees from four organizations to examine job satisfaction and wellbeing experiences in the workplace and potential strategies for supporting health and wellbeing of people with an intellectual disability.

**Results:**

The findings revealed that currently there is a strong emphasis on strategies such as mentoring and support, flexible approaches, and customized and varied roles to support mental wellbeing. There seems to be less focus on physical activity and nutrition with limited examples of strategies addressing these topics. There are also instances of bullying being experienced in open employment settings.

**Conclusions:**

Further work is required to verify whether these results are consistent across the sector, but it does seem to illustrate that workplace wellbeing intervention models and strategies that are applicable in workplaces for the general population may not necessarily work in employment settings that are inclusive of people with an intellectual disability. The policy implication is that support structures so important to health and wellbeing within supported employment settings also need to be available in open employment environments. Further research and policy work is required to develop specific models and strategies that will be applicable to this population cohort within supported and open workplace settings.

## Introduction

People with intellectual disability represent an important population group for public health attention. Worldwide estimates have the prevalence of intellectual disability at 1% (1) and in Australia the prevalence varies between 1.6% and 2.7% of the population depending on which measurement criteria are used (2). People with intellectual disability have higher rates of diabetes (3) and obesity (3, 4) relative to the general population, have lower levels of physical activity, less healthy diets, (5–7) and an increased rate of mental health problems (8). While the needs are greater, this population cohort are less likely to be the focus of health promotion research and interventions (9, 10).

An important setting through which health and wellbeing can be addressed for people with intellectual disability is the workplace given the significant numbers that are employed (11). While precise numbers are difficult to ascertain, data from 2022 indicated that around 16,000 people were employed in what we deemed supported employment (previously labeled as Australian Disability Enterprises, ADEs) (12). There would also be many people employed in open employment settings although not to the extent recommended by the Disability Royal Commission (13). They considered that supported employment still represents a risk setting for exploitation, violence, and abuse (13). Presently the rate of transition between supported and open employment is very low both in Australia and internationally varying between 1 and 4% for different age groups (14, 15). The commission recommended that open employment settings should be the default option for school leavers and some commissioners were of the view that supported employment should be completed phased out (13). Whether people with intellectual disability work in open or supported employment contexts, there is considerable potential to promote health and wellbeing in these settings (11).

Amongst the general population there is good evidence that workplace health promotion programs encompassing organizational change can positively impact a range of health and wellbeing topics including mental health, nutrition, physical activity, and can reduce absenteeism (16–21). While education only interventions have shown to be less effective (16, 21), those interventions that encompassing cultural and organizational change strategies have a good evidence base (16, 18, 20, 22). Multi-component programs including both individual and organizational strategies are referred to as comprehensive or socio-ecological approaches to settings based change (23). One of the significant gaps in workplace health promotion research is intervention models for different population groups (22), including a gap in addressing the workplace health promotion needs of people with an intellectual disability (11).

At present, there is very little research on how to promote wellbeing in the workplace for people with intellectual disability (11). There has been some research on job satisfaction amongst people with intellectual disability and this research has uncovered the importance of social connections and support which align with key concepts in public health and health promotion (11). This research is predominantly quantitative and there has been recommendations for more qualitative research so that a richer understanding can provide a guide to designing intervention and workplace health and wellbeing models (24). This paper reports on a study conducted to understand how people with intellectual disability experience both supported and open employment with respect to job satisfaction and health and wellbeing. The study includes analysis of in-depth interviews that were conducted with 47 people (16 supported employees, 24 staff and managers, and seven external stakeholders). The questions focused on some of the health and wellbeing experiences at work and the factors influencing job satisfaction and health and wellbeing. The results confirmed previous research on the importance of social connections and support but also illustrated other themes including the importance of variety of roles, and tailoring roles. There were also some interesting observations on the risks posed in open employment settings which have policy and further research implications discussed in the paper.

### Job satisfaction and health and wellbeing of employees with an intellectual disability

While there has been little health and wellbeing research in workplaces with people with an intellectual disability, there has been some work on understanding the predictors of job satisfaction. A range of quantitative studies have found that higher levels of job satisfaction are related to a sense of autonomy, strong connections, and a sense of competence (25). A recent study involving 554 workers from Spain found that increased job satisfaction was related to lower psychological demands and reduced exhaustion (24). Higher levels of job resources (such as support from co-workers) has also been related to increased job satisfaction in a number of studies (24, 26, 27). In the Flores et al. (24) study, the single biggest predictor of job satisfaction was support from supervisors and support from co-workers was also an important factor.

Flores et al. (24) also compared between sheltered employment (which would be similar to the ADEs in Australia) and what was termed inclusive employment in the community. The results generally showed that all participants had high levels of job satisfaction but there were group differences. Those in the inclusive employment settings did have higher levels of job satisfaction, work engagement, lower overall scores on job demands, and increased scores on job resources. This is in contrast to other research which has not found differences in job satisfaction between the different types of employment options (26). One of the conclusions from the Flores et al. (24) study was that social connections may be an important focus for wellbeing and job satisfaction interventions but there would need to be further qualitative research to understand other important factors influencing job satisfaction for people with an intellectual disability.

There has been some qualitative research on wellbeing in the workplace for people with an intellectual disability from a Human Resources perspective (28). This research highlighted the importance again of supervisor and co-worker support and connection for wellbeing (28). Further to this study, another research project examined the role of managers in implementing wellbeing initiatives for employees with an intellectual disability (29). Some of the important factors according to these managers were “checking-in” with employees and the need for an empowerment and partnership approach. There is a need for more qualitative research on important factors for wellbeing and inclusion within the workplace for people with intellectual disability, particularly comparing organizational types. Previous research has suggested that there may be differences in job satisfaction and wellbeing indicators between more community focused, compared to segregated employment settings. Further research is required to understand in-depth the experiences of people with intellectual disabilities across different organizations and how their health and wellbeing can be supported across all employment settings.

The aims of this article were to examine the experience of job satisfaction and health and wellbeing across different types of workplaces and some of the factors that determine these health and wellbeing outcomes. The research questions were:

- What do people with intellectual disability and support staff consider are the important factors in health and wellbeing in the workplace?
- What experiences do they have with any health and wellbeing strategies and what are their perceptions of those strategies?
- Are there different health and wellbeing experiences in different workplace settings? Particularly between supported and open employment?

The research questions were guided by a socio-ecological settings perspective (23), which has the strongest evidence base in workplace health promotion. This research can then provide a platform for intervention studies seeking to improve job satisfaction and wellbeing amongst people with an intellectual disability.

## Method

The data analyzed comes from a project funded through the Information Linkages and Capacity Building Scheme of the Department of Social Services. The aims of the research project were to examine the specific features of supported employment organizations that were able to successfully transition people into open employment opportunities. Part of this research agenda focused on examining job satisfaction and wellbeing across supported and open employment of people with an intellectual disability.

Four organizations participated in the study. Each organization provided a range of employment options within their organization, from more traditional supported employment typical of disability enterprises to more community facing roles but still with a range of job supports provided. Each organization had examples of where people had transitioned to open employment. Forty-seven semi-structured interviews were undertaken with participants to understand the different experiences of job satisfaction and health and wellbeing across different organizations (16 supported employees, 24 staff and managers; and seven people who were either from partner organizations or family members). One organization had been involved in the project for 3 years and the other three organizations only 1 year. Most of the data collection has taken place with organization 1 (33 interviews), seven interviews from organization 2, five interviews from organization 3, and two interviews from organization 4.

The inclusion criteria for an interview were staff and managers of people in the workplace, employees with an intellectual disability that had experience across both supported and open employment, family members, and open employers with experience of employing people with an intellectual disability. People were informed about the project during team meetings and were told to contact their manager if they wished to participate. Participants were provided with a Plain Language Statement which was also available in Easy English and if required, the information was read to participants. The questions for people with an intellectual disability focused on what they liked and did not like about their job, what made them happy in the workplace, and any differences between different jobs they have had. The interview questions for staff, partner organizations, and family members focused on what they considered were the important factors that influenced health and wellbeing in the workplace.

Given the limited research on health and wellbeing in the workplace for people with an intellectual disability, there was no particular analytic frame that could be used and hence a thematic analysis was undertaken (30). According to best practice qualitative research, each of the data collection and analytic steps has been described (31). Researcher triangulation was used to reduce bias and confidence of the findings whereby two researchers were involved in conducting the interviews and coding the data (32). The data was first analyzed using thematic analysis to uncover themes related to job satisfaction and health and wellbeing in the workplace (33, 34). This involved a process of reading the data line by line and grouping the data into meaningful categories (35, 36). The data was also analyzed to examine differences and similarities between experiences in supported vs. open employment (37). The next analysis stage involved drawing connections between the key themes (36) and how these different organizational types and characteristics influenced job satisfaction and health and wellbeing outcomes. These themes were discussed and refined with all study authors. As a final credibility and integrity check, the themes of the study were presented and discussed in a meeting with two staff representatives from each of the four organizations who had been part of the steering committee of the project (31).

### Ethical consideration

The study was approved by the Human Research Ethics committee of Swinburne University of Technology.

## Results

The results are presented according to the key themes of job satisfaction and wellbeing experiences of people with an intellectual disability. The majority of the results relate to experiences in supported employment settings. The results begin with the some of the positive experiences related to support and connection, and the provision of tailored and holistic support. The next themes presented are also positive and relate to having a sense of purpose, experiencing a variety of roles and workplace locations, and tailoring the role to each employee. The results also cover areas of health and wellbeing that are receiving less attention in physical activity and nutrition. The final section of the results contrasts the experiences in supported employment with experiences in open employment settings. It is revealed that some of the same health and wellbeing experiences were encountered in open employment settings but there were also some negative experiences particularly related to lack of support and in some cases, bullying.

### Strong sense of support and connection

One of the most important factors in job satisfaction and wellbeing at work was the relationships and support with colleagues and support staff. Critical to this was feeling respected and part of the team. Feeling valued and a strong sense of connection was important to the employees that were interviewed:

“Yeah, that's the kitchen. It's really good when you work in a place full of people that are really nice and helpful and give you nice sayings and stuff like that.” (Organization 1, Supported Employee 1)

“Respect is like you can do a job and someone can let you have the right amount of time to do the job, or they can give you the right equipment. They don't put pressure or stress on you…They're friendly and kind.” (Organization 1, Supported Employee 11)

“But a lot of them want to stay here because it's where their friends are, they feel safe.” (Organization 1, Staff 9)

This last quote is in reference to staying at a supported employment context rather than moving to open employment. Some of the challenges with moving to open employment will be covered later in the results with people feeling that there was less social connection in open employment settings. Part of the strategy of ensuring a sense of wellbeing in the workplace was ensuring that people felt connected and enjoyment at work and that they were treated like all employees:

“They're excellent here...You're not looked at or judged like you have a disability here.” (Organization 1, Supported Employee 2)

“Having fun at work as well. So, we do have a fair bit of banter on the floor. I'm usually called a smart arse and they always like to give it to me. I think that's because I speak to them and treat them the same as I would at all my other jobs and all my other employees. So, we'll tell jokes.” (Organization 1, Staff 6)

There were several different strategies that the organizations used to provide a sense of support at work. Some organizations used a buddy or mentoring system to provide initial orientation and support in the workplace. In some instances, this was provided by supported employees with more experience in the workplace, or alternatively this was provided by support staff:

“And what we do is we use peer mentoring with some of the new supported employees. So the new ones who come on will use the ones who've been there for ages as a mentor and they feel really valued in being able to teach their learnings to somebody new. So things like that make a really good difference.” (Organization 2, Staff 17)

“We support them and everything, but we're becoming more like an open employment style business because I'm getting more staff and they're working side by side, rather than, ‘Let me train you and here's a job and I'll just supervise and watch while working side by side'.” (Organization 1, Staff 5)

In addition to mentoring and buddy systems there were also examples of service type support provision. One strategy used to provide support was through the provision of employment counseling which was considered important in supporting mental wellbeing:

“I think mental health of our clients is a much bigger factor than I ever thought it was, and having our employment counseling as core to our service, I feel, is extremely valuable…So having counselors that can go into the workplace, that know the person really well, that can communicate with the person and support the person to self-advocate within an organization, is huge for that person's integration and mental health in the workplace.” (Organization 3, Staff 21)

Another key strategy for providing social support was ‘checking in' either informally during the day or in more structured ways through meetings. This was seen as an important strategy for supporting mental health and being proactive in addressing any issues that might arise:

“I guess checking up on them, making sure that they're okay and that they have a say and they're contributing to what's happening in the workplace as well to make sure that they feel valued as an employee. What we do is we have morning huddles, we call them. So it's pretty much having a little bit of a team meeting at each of the social enterprises and talking about what's happening for the day. And it gives everyone an opportunity to touch base, see what they did over the weekend and just have a little bit of a brief catch-up.” (Organization 2, Staff 17)

In addition to these group catch ups, this organization also had opportunities for one-to-one meetings with the supported employees each month and quarterly reviews with their goals which provided other opportunities to check up on their wellbeing at work.

While the majority of interview participants commented on positive relationship aspects with the supported employment context, there was acknowledgment of challenges with social relationships that had to be managed:

“That's definitely one we look out for and also just keeping an eye on how their relationships are going with other people in the workplace, because sometimes there can be conflict and that can obviously impact them.” (Organization 1, Staff 2)

Having a workplace with multiple sites and work roles was viewed as helpful in ensuring that people could be moved to avoid certain conflicts and being able to place people that connected well together.

“So, the old chef that was there, I got along with her really well, and then the new chef came in and we butted heads a bit, and then I was like, ‘I can't be in the kitchen anymore…Yeah, and the chef was stressing me out, and I was like, ‘Oh, no'.” (Organization 2, Supported Employee 12)

One of the advantages of these case study organizations were that they were large enough with different sites and business operations that they could move people around if there were relationship conflicts that needed to be managed. This flexibility in locations and roles was also important in itself for wellbeing and job satisfaction which we will be addressed later in the results as it provided confidence that people can adapt to different circumstances. Variety of locations was also helpful to manage the social dynamics to ensure that people felt connected and supported within their workplace setting and they could be shifted if this situation changed. This helped to build resilience amongst supported employees.

### Tailored and holistic support

While support staff at the organizations were conscious of treating everyone as equals from a social connection and team member perspective, in regard to specific supports there was a tailored and holistic approach adopted. They could provide support around service coordination, accessing health services, supporting with mental health, connecting with family members around any apparent issues, and ensuring people have access to lunch if they forgot food:

“They may not have support coordination. They may not have some core supports in their plan … There's a gap. And our team do fill that. They assist with doctors' appointments. They've been assisting people with vaccination appointments and getting prepared for that … So, there's things that we do over and above … Our core purpose about having a positive impact on people's lives is very much ingrained.” (Organization, Staff 1)

“Well, first of all, we make sure that people have got lunch, and if they don't, we ask why … We do welfare checks, like if there's somebody that hasn't—somebody reaches out to me regularly, but they haven't for a while, so I will call to make sure they're all right.” (Organization 1, Staff 16)

There is a culture within these workplaces of providing a level of support well beyond what would be expected in an open employment context. People are supported with aspects of their life outside of just their work role:

“It's not just about the employment, it's looking at those other wraparound supports for them in the morning before they come to work and things like that.” (Organization 2, Staff 17)

Some of the organizations provide employment supports alongside other services such as supported living which makes coordination across services easier. But all employment providers had networks across different services to ensure that wellbeing was addressed holistically both in and outside of the workplace. This meant that support staff were constantly attune to issues that might be arising outside of work and how they could potentially assist where required:

“If someone comes in late every day, there could be other issues. We try and look at everything. It could be an issue where they've run out of NDIS funding, and they're walking to work …Everything can lead into something else, and that's what I tell my team. We always try and think, especially changing behaviors, what's going on?” (Organization 1, Staff 3)

“And informally, they're [disability enterprise supervisors are] tuned in to the precursors for anything that might happen; if the client's having a down day, or their mood is low, or whatever. And then can…change work roles around, so there's a lot of flexibility. And informally, they might talk to the client around, hey, maybe you need to go home and just rest today and then come back tomorrow, or whatever. So that's the essence, or one of the crucial essences of supported employment.” (Organization 2, Staff 18)

Thus, part of the sense of job satisfaction and wellbeing at work is created by a culture that supports the overall health and wellbeing of employees. This means addressing all issues that could arise both in and outside of work and putting in place a range of supports to address these issues. Often this relates to mental health but as seen through the data it can also relate to medical issues such as medication and issues arising in supported living and other settings in their lives. This highlights the need for a coordinated approach to wellbeing across multiple settings in which people live and work. It also again illustrates the importance of building strong social connections where a culture of trust and support is developed as illustrated in the first theme. This provides the foundations for being able to respond holistically across multiple domains of a person's life as the setting is viewed as more than just a workplace. The last quote also references flexibility in roles which will be addressed in the next theme.

### Variety of roles and locations

Having a variety of roles was viewed as another element that supported wellbeing and job satisfaction. This meant that people could choose from different roles to suit their particular skills. It also meant there was variety so that people were not stuck doing the same task every day and there was rotation in the roles they were performing. This was seen as important for mental health and wellbeing alongside the flexible culture that was previously mentioned. The same wording of liking a “bit of everything” was used by two different people including the following quote:

“If I want to go to do cooking, then I can talk to and they can help transfer me from the nursery and they'll get me into the cooking… I like a bit of everything.” (Organization 1, Supported Employee 6)

“I'm pretty much an all-round person here…Yeah, there's always a lot of different jobs here, and that's what I've done today, I've been round everywhere today. Which is good, because it makes the day go quicker, and you're never bored…And here in hospitality, it's a job that I can actually do, and I'm really proud because 2 years ago when I had to give up the farm work, I thought I was never going to work again in my life.” (Organization 1, Supported Employee 2)

The person who made this last quote also commented on the extra support that is provided in this organization compared to open employment and that as a trade-off they were happy to have less pay relative to open employment given the extra social support available. While the pay is less, when considering the combination of both the pension and this income, this interview participant felt that was sufficient in meeting their needs. Again, a contrast was made with the lack of support provided in open employment.

Being able to provide a variety of roles was contingent on organizations having a variety of business streams, multiple sites, and different jobs within each of these locations and businesses. Supported employees are encouraged to attempt different roles and move to different locations so that they develop new skills and develop resilience to cope with change. This was seen as important in building confidence to attempt open employment if that was a goal. Related to the benefit of having different locations, was having different workspaces. Some spaces were very social such as a café environment, but there were also areas of quiet and solitude. Having a variety of workspaces and areas where people could relax was seen as important for mental health and wellbeing:

“And you just go to the supervisor, ‘Can I just have some time out?' And they understand, ‘Okay, all right'. Then they'll put you somewhere, just quietly on your own and you'll just need some chill out time. Because sometimes you just want to work on your own.” (Organization 1, Supported Employee 4)

“When we have break, any free room, any free function room, we just go in. Sometimes I go to the reception if there's some people in those ones. I'll just go, well, what rooms are free, and there'll be a room, and it's great because they're dead quiet and you just sit in there.” (Organization 1, Supported Employee 3)

Being able to provide a holistic style of support was contingent to some extent on having different areas and spaces that supported employees could use during the day.

### Task modification and purpose

Another key element related to job satisfaction and wellbeing at work is modifying work roles and work conditions to suit people's abilities while still ensuring that there is a clear purpose to the role. All the case study organizations were able to change the layout of spaces and provide accessible equipment to ensure that people could perform work roles. These involved large scale adaptations related to furniture and machinery but also small changes. The following example involved laminated instruction cards to assist a supported employee:

“She's put sheets out for me to know the basic stuff to read which is a café sandwich, or a basic sandwich or a wrap that comes to the till. With the coffees, there's buttons to press, she's put a sticker on the buttons for small and large … And it does make it more enjoyable, and more of a happier workplace. But it also makes it an efficient workplace as well.” (Organization 1, Supported Employee 2)

It was previously mentioned that variety of roles was important for wellbeing. However, for some participants their level of ability required a more specialized role, and this is an example of creating a position to suit the needs of a supported employee:

“We have one guy that, all he does is stamp a number seven onto a brown paper bag, which is part of our assembly line. And loves it. Super excited coming to work. Absolutely loves it. So we've job carved out that one step of that, just for him.” (Organization 4, Staff 24)

Whatever the role, a sense of purpose and dignity to the position was of paramount importance. While income is clearly important and there is considerable debate about award wages within the industry, a sense of purpose in the role was the key factor according to a number of participants:

“I take a lot of pride in what I do. I'm not somebody who comes to [organization] and just goes through the motions and say, pay me…the money doesn't motivate me. The pride motivates me. And receiving good feedback from the people who are working for me or are working with me. To receive good feedback from them is the icing on the cake for me…To know that they can rely on me to do a good job, which means they don't have to look over my shoulder.” (Organization 3, Supported Employee 15)

“And he's now in a residential and on a huge NDIS plan, but in his head, he's still working at day programs and he's still earning money, when he goes to day programs, he's going to work. That's what he calls it. He's not earning anything. Because to him—and he helps them with tasks, so they go out and they do gardening and they go to the workshop and they do work, and he helps clean the center and things like that. But that's his day program, and that's giving him a sense of fulfillment and contribution to society, which is huge for mental health and wellbeing. And I think that's missed when people say what someone's earning. They're forgetting about the sense of empowerment and achievement and the social side that comes from work and having a structured day.” (Organization 1, Staff 13)

“It needs to be something that they know we need to have happen and then they know that they're contributing and they know that it is well appreciated, that what they're doing is a good job.” (Organization 2, Employer 1)

This study did not seek to investigate perceptions around supported and award wages and it is not within scope to be making commentary around research or policy suggestions on this topic. However, these quotes highlight the significance of purpose and social connection in the role. It matters less whether it is supported or open employment, or in some cases through day programs or voluntary, where organizations are providing a sense of purpose and fulfilling the social needs of the person, this is what is important for people's mental health and wellbeing.

### Physical activity and nutrition

In reflecting on the broad definition of health and wellbeing, the results have mainly focused on mental and social wellbeing. The mental health and social wellbeing elements were the main topics that people felt were relevant to job satisfaction and were the core focus of the case study organizations. However, our research indicates that physical activity and nutrition are equally important health topics for people with an intellectual disability and workplaces have an important role in addressing these topics. In regard to the case study organizations there was some acknowledgment of physical activity and nutrition, but it was apparent they were not high priorities.

Some supported employees do have roles that inherently involve some physical labor as part of the role, such as gardening. For other supported employees, there seemed to be less focus on physical activity itself as a goal within the workplace unless it related to a mental health focus. For example, the following supported employee used basketball as a coping mechanism when feeling stressed within the workplace:

“The support worker was always checking in to go, “Mate, you're all good.” Do you need go out and play basketball,” because he loves basketball. When he was worked up, they used to just go out and shoot hoops, have a chat, and come back in which was really useful as well in that space.” (Organization 4, Staff 23)

In this instance physical activity in the form of basketball was used to help manage the anxiety of a supported employee as he attempted the transition to a more community facing role. Again, with nutrition there was some acknowledgment of the topic but it did not seem to be a priority with any of the organizations. One respondent initially felt they were not doing any work around nutrition but then remembered some strategies:

“I'd have to say no but now I'm thinking in my head it shouldn't be no…I do know in the café actually now I'm thinking about that, that they do have picture cards and I guess color coded foods... But as far as themselves being supported and educated around diet and physical activity, I don't think so.” (Organization 4, staff 23)

Another interview participant elaborated how they did cover physical activity and nutrition but that it was not an organizational priority, and a sense of choice and empowerment was fundamental to the approach taken:

“But there is a bowl of fruits if you want to have that, but what we work on is your wellbeing and your self choice and your independence to actually be able to choose—yes, we'll let you know in the mornings, “Here's the healthy foods, here's the non-healthy foods,” and discussions around that. We also do some kind of physical activity every now and then, but it's not something that is regularly a part of our operational approach…we talk about the different choices that they have, and encourage them to make the right choices.” (Organization 3, Staff 20)

Being able to provide the appropriate nutritional environment is considered a challenge and this example highlights some of the tensions experienced in an open employment context:

“One of the bad things with our work experience is that we do a lot of [organization names] and places like that, and they like to leave treats and that for their staff in the staffrooms. Often, we'll find them, if they've been gone for a while and we have to go look for them, we'll find them sitting at the staff table eating a Milo bar or a Crunchie, or a lolly, or a bag of chippies that they've put out. Which is fine, because the managers say, ‘Help yourself to that stuff during your breaks.' But they kind of like do it during an unscheduled break.” (Organization 1, Staff 16)

Offering a more personal example, one staff member commented on needing to buy lunch for supported employees if they ever forget. While the sense of support is the key point of this quote it also reveals that thoughts around nutrition are secondary as the cheapest food in a food court is very unlikely to be nutritious:

“I couldn't let anybody go hungry. Even if you go buy them the cheapest food in the food court.” (Organization 1, Staff 16)

The results illustrate that nutrition and physical activity were addressed to some extent within the various case study organizations and people were aware of these as important issues, but they were not high priorities. Further, there was some tension between being able to provide healthy options but also ensuring that people had choice and control over their daily activities and food preferences.

### Open employment experiences

The last theme concerns the health and wellbeing experiences of supported employees who were transitioning to open employment. In the supported employment settings, the previous results illustrate a number of factors that were important to health and wellbeing. These included social connection, holistic support, task modification, sense of purpose, and variety of roles. The final section of the results covers whether these factors were also experienced in open employment settings.

In open employment there were some positive instances of the same type of tailored support and strong relationships that are viewed as so critical to wellbeing in the workplace:

“I think the first thing we should do is spend a fair bit of time getting to know each other so they feel confident they can trust us and we know this person and where they're at and what their needs and abilities are. So we're not setting the bar too high or nor are we being placatory, we're actually treating them respectfully.” (Organization 2, Employer 1)

“That was quite similar where I would get a list on what needed to be done for the day, and it was like, ‘Here's the folder of all the recipes, just go through them with that.' That was a little independent as well. I was doing the wholesale stuff, so the stuff that would go to other cafes and that, so that was quite good.” (Organization 1, Supported Employee 3)

One of the interview participants was able to provide two examples of open employers providing a similar level of social and practical support to employees with disabilities that would be expected in a supported employment context:

“There's one employer that we work with, they have a morning meeting every morning and they go, ‘Let's rate how we're feeling one to ten?' And they bring everyone together, all the employees. And there's two people out of that group that have a visible disability; one's got intellectual and the other one has down syndrome, similar…That's really important for them to actually have that touchpoint and going, ‘Where's everyone at today before we get going in our day.' They found that as a workplace really good to know where everyone's at and they found that it's opened up more conversations with each other to help and support.” (Organization 4, Staff 23)

Some organizations were able to provide very clear instructions on who people could ask for help and that they could swap roles between customer service and back of operation depending on how they were feeling:

“They [organization] were very open in relation to adapting a flexible model, and that's why they only had key people on the floor to go, ‘You know what you can speak to these three people today,' And they had pictures of them in the staff room if they've got any questions, concerns or troubles.” (Organization 4, Staff 23)

These positive examples of the same level of support and adjustments being made in open employment, were accompanied by feedback where this was not the experience. People commented on feeling unsupported in the workplace and not being accommodated in a respectful manner and that impacted negatively on their self-confidence and worth:

“I couldn't carry heavy pots and pans and because I couldn't do a lot in hospitality like carry the heavy pots and pans and all they did was tell me to stay out of the way you can't go near the stove or the oven. It was like they're showing me, like I'm some special disabled person. And I hated that because I don't want my disability to affect my job. I just want it – I know it's there.” (Organization 1, Supported Employee 11)

“Yeah, they weren't very nice to me…Like I would mow half the lawn and the catch wasn't down. So no-one helped me. And then I was doing all that, and they go, ‘You didn't put the catch down, so you have to do it all over again'…they just weren't very good. It was horrible. I didn't like it. And when I had lows, they didn't look after me that well.” (Organization 1, Supported Employee 7)

“I worked at [organization] and I was a cashier and I thought I was selling the products but I was on cashier, which is not my strength, with money. And I was only working there for 2 weeks. They'd showed me one time how to use the till and how to work the till and stuff like that, but because I have short-term memory loss I work by showing and myself doing it and they didn't give me much help.” (Organization 1, Supported Employee 1)

As well as a lack of support being provided, one of the other problems identified was a lack of variety and that the work could become monotonous:

“Variety of work, when we've had people go into open employment and come back, it's because they're bored doing the same thing every day, whereas here we've got variety and upskilling. So, they see that as a barrier.” (Organization 1, Staff 6)

Lastly, there were instances of bullying in the workplace that were reported:

“She said she worked in a hospital doing the tea lady stuff and that. I went, ‘Oh, that would have been really lovely.' And she goes, ‘I just got bullied.' But she was there for years before it got noticed. I'm just like, oh. And they get taken—I'm sorry—they get taken advantage of.” (Organization 2, Staff 19)

“The workplace was not inclusive. They employed her originally and completely well aware of who she was, her disability, her barriers, and her limitations but the employer, it was like a café shop. But when the main person wasn't there, she was very badly bullied, she was told to get in a sink, that's where she belongs. Very discriminated against, laughed at, had food thrown at her, things like that. It was horrific, absolutely horrific. And before, she was putting up with this for nearly 2 months before she told anyone, and that was to the point that she just burst out in tears and refused to go to work.” (Organization 4, Staff 23)

As part of their response to this instance of bullying and discrimination, the staff from the supported employment organization helped this person to get a position back in supported employment and are now in the process of trying to build her confidence up. This last quote was the most extreme case of bullying uncovered through these interviews, but the lack of support in open employment was a consistent finding. Interview participants mentioned that many supported employees are reluctant to move into open employment due to concerns about lack of support, while a number of people have moved back to supported employment due to negative experiences. Many of the positive experiences of supported employment such as variety, holistic support, task modification, and social connection, were not experienced in open employment roles.

## Discussion

The results of this qualitative study confirm some of the key findings from previous quantitative research on job satisfaction of people with intellectual disability in the workplace. These include the importance of social connections and social support (24, 26, 27). A sense of connection with peers was seen as critical, while emotional and practical support from supervisors was also important for wellbeing and job satisfaction. The skill in providing this support was ensuring that people felt valued in their roles and empowered. The findings of this research also corroborate other qualitative research which highlights the importance of processes like “checking in” with employees and other supportive practices for mental health (29). These findings can help address a research gap in resources for organizations on how to support wellbeing in the workplace for people with an intellectual disability (11). A practice guide can be developed on supporting workplace health and wellbeing for people with intellectual disabilities which includes these elements.

The results provided some detail beyond the existing quantitative research on other factors that may be influencing job satisfaction and wellbeing among people with an intellectual disability. This included having a variety of roles and a variety of different spaces within the workplace. These different spaces enabled people to have quiet times and socialize depending on their preferences. These themes illustrate how organizational design of spaces can influence the experience of wellbeing in the workplace (38, 39). Again, it will be important that any guide for promoting wellbeing in the workplace for people with an intellectual disability highlights the importance of these factors. Each organization would have different ways in which they can address factors such as the use of space and variety of roles depending on industry and organizational focus. Thus, any practice guide would need to provide direction without being overly prescriptive on how each element is addressed.

These findings illustrate that the case study organizations were highly focused on the social and mental health dimensions of wellbeing. Physical health behaviors, particularly nutrition and physical activity seemed to be less of a priority. The research shows that people with intellectual disability are less physically active and have less healthy diets relative to the general population (5–7), and have higher rates of diabetes (3) and obesity (3, 4). However, these issues can be successfully addressed in the workplace (20, 21). There is further intervention research required on how to address nutrition and physical activity within the workplace with people with an intellectual disability.

The findings of this study have relevance for addressing job satisfaction and wellbeing within social enterprise (businesses with a social purpose that also trade for a profit) and supported employment contexts. However, there were also some interesting observations on open employment experiences. While there were some encouraging examples provided of support and connection within open employment contexts there were also some negative findings. The primary aim of this research was to understand positive examples of supporting wellbeing and job satisfaction so uncovering negative experiences was not a focus. This is a limitation of the research which will be further acknowledged in this section. However, there were a few stories of poor experiences that impacted negatively on wellbeing, particularly related to bullying. There is considerable research illustrating that bullying in the workplace is related to various measures of mental health including depression, anxiety and psychological distress (40). Workplace bullying has also been linked to an increase risk of diabetes (41), and diabetes is an health problem that people with intellectual disability are already at higher risk of experiencing (3). Bullying is clearly an issue of importance within the workplace.

There is little research on the experience of workplace bullying among people with intellectual disabilities and disabilities more generally (42). There was a UK study that investigated incidence of bullying and other negative experiences at work which they labeled as ill-treatment (43). People with disabilities experienced a higher rate of these negative experiences including bullying. The authors developed three categories of bullying and included learning difficulties in the same group alongside psychological, and emotional problems. Relative to the two other disability groups (physical and unspecified) this group had the highest reports of negative experiences. The authors recommended that in future research when performing the survey and statistical analysis, people with learning difficulties are grouped separately. There has also been an intervention study to reduce bullying frequency within supported employment settings in the UK (44). This involved peer to peer bullying and the results showed that 43% of people had experienced bullying and 28% reported being a perpetrator prior to the intervention commencing. The intervention was successful in reducing these frequencies. There seems to be little research we are aware of examining and intervening in bullying between staff with and without intellectual disability. Given the power differential and possible social exclusion, this type of bullying requires further research, (43) to examine its prevalence in different employment contexts.

More broadly, the findings do corroborate previous research comparing the experience of employees with an intellectual disability across sheltered, social enterprise, and open employment (45). Similar to the results presented in this article, that study found that people experienced discrimination in open employment (45). The previous research found that social enterprise provided the combination of both high levels of support and community integration whereas open employment provided community integration but less support, and sheltered employment provided support but lacked community integration (45). The policy ambition in Australia that open employment is the first option considered for people with an intellectual disability, is also an aspiration of many developed countries (13, 46). As covered in the introduction, the Disability Royal Commission considered that supported employment is a potential risk setting for exploitation, violence, and abuse (13). This article highlights some of the potential risks in open employment. Supports and social connection that were present in supported employment may not be available in open employment and there is a risk for bullying. Further research is required in determine the extent to which people with intellectual disabilities experience bullying and other negative experiences in open employment. The policy implication is that caution and careful evaluation is required to track the full implications of increasing transition to open employment.

This article highlights what type of workplace experience is required to support job satisfaction and wellbeing for people with an intellectual disability. The important factors are strong social support, community connection, variety, and flexibility. Presently, this type of work is most commonly associated with social enterprise, and considerable reform is still required of open employment and what used to be termed sheltered employment (45). The findings of this research can help guide reform of the supported employment sector as they transition to social enterprise and also for open employers with employees that have an intellectual disability. Policy and practice guides can be developed to ensure that all workplace settings provide an inclusive and supportive workplace environment.

This study has been able to provide more in-depth findings about the factors that support job satisfaction and wellbeing among employees with an intellectual disability. It has also highlighted areas requiring further analysis such as nutrition and physical activity in supported employment contexts, and bullying in open employment. The limitation of the research was the small sample size which hindered the ability to generalize beyond these case studies. The purpose of the research was to ascertain best practice examples that could help guide future intervention research. Further research is required, however, to determine the extent to which the work conditions uncovered in this study are experienced more broadly. Future research could also involve people with intellectual disabilities as co-researchers which was a limitation of this research project. Including people with intellectual disabilities in a co-design research process will be important for intervention research (11). Another limitation was the focus on positive aspects of wellbeing and work conditions more so than negative experiences, although the research did uncover problems in the workplace as described. Also, the majority of the interviews took place in one organization which limits generalizability. While these are the major limitations of the research, the findings are consistent with similar research which increases confidence in the findings (45).

## Conclusion

This study has revealed some of the important elements influencing job satisfaction and wellbeing among employees with an intellectual disability. These key factors are high levels of emotional and practical support, variety in job roles, variety of locations and different spaces, integration within the community, and a clear sense of purpose for the role. The research also uncovered some negative experiences within open employment such as bullying that requires further research. These findings can now be used to help design workplace wellbeing interventions for people with an intellectual disability which is a current research gap (46). Health promotion workplace intervention models for the general population are not necessarily applicable to this cohort, as their needs are slightly different particularly with respect to the type and level of social support required. This research has provided a blueprint for a health promotion workplace model for organizations employing people with an intellectual disability. The results can also help shape current reforms to increase employment rates of people with an intellectual disability as they provide a guide to the type of employment experience that enables people to succeed in their roles. Applying this research at a policy and practice level could achieve the twin benefits of promoting health and wellbeing and also increase employment opportunities. Further intervention research is required to help realize these ambitions.

## Data Availability

The raw data supporting the conclusions of this article will be made available by the authors, without undue reservation.
